# Association between extreme heat and hospital admissions for cataract patients in Hefei, China

**DOI:** 10.1007/s11356-020-10402-1

**Published:** 2020-08-13

**Authors:** Jingui Xie, Yongjian Zhu, Yiming Fan, Linbo Xie, Ruijin Xie, Fengming Huang, Liqing Cao

**Affiliations:** 1 School of Management, Technical University of Munich, Bildungscampus 9, 74076 Heilbronn, Germany; 2 School of Management, University of Science and Technology of China, 96 Jin Zhai Road, Bao He District, Hefei, 230026 Anhui People’s Republic of China; 3 Anhui Health College, 9 Xue Yuan Road, Jiao Yu Yuan District, Chizhou, 247099 Anhui People’s Republic of China; 4 The First Affiliated Hospital of University of Science and Technology of China, 17 Lu Jiang Road, Lu Yang District, Hefei, 230001 Anhui People’s Republic of China

**Keywords:** Extreme heat, Hospital admission, Cataract, Generalized additive model, Distributed lag nonlinear model

## Abstract

Cataract is the first cause of blindness and the major cause of visual impairment worldwide. Under conditions of global warming, researchers have begun to give attention to the influence of increasing temperature on cataract patients. Our paper aimed to investigate the association between extreme heat and hospital admissions for cataract in Hefei, China. Based on data from the New Rural Cooperative Medical System and National Meteorological Information Center, we used a generalized additive model and a distributed lag nonlinear model to examine the relationship between extreme heat and hospitalizations for cataract, with consideration of cumulative and lagged effects. When current mean temperature was above 28 °C, each 1 °C rise was associated with a 4% decrease in the number of cataract admissions (RR = 0.96, 95% CI = 0.94–0.98). The cumulative relative risk over 11 days of lag was the lowest, which indicated that every 1 °C increase in mean temperature above 28 °C was associated with a 19% decrease in the number of hospital admissions for cataract (RR = 0.81, 95% CI = 0.75–0.88). In subgroup analyses, the negative association between extreme heat and hospital admissions for cataract was stronger among patients who were not admitted to provincial-level hospitals. In conclusion, this paper found that extreme heat was negatively associated with cataract hospitalizations in Hefei, providing useful information for hospitals and policymakers.

**Electronic supplementary material:**

The online version of this article (10.1007/s11356-020-10402-1) contains supplementary material, which is available to authorized users.

## Introduction

Cataract, the opacity of lens in eyes, is a common disease in the elderly, and patients could notice it at an early stage (Anderson et al. 2018; Prokofyeva et al. 2013). Currently, cataract is the first cause of blindness and the major cause of visual impairment worldwide (Pascolini and Mariotti 2012). In China, the highest prevalence of cataract occurs in the south-central part of this country, possibly caused by the long-term exposure to higher ultraviolet radiation (Delavar et al. 2018; Modenese and Gobba 2018; Prokofyeva et al. 2013; Song et al. 2018). It is predicted that the number of people affected by cataract in China will be 240.83 million by 2050 (Song et al. 2018). Therefore, the management of cataract patients has become a significant public health problem.

Since 1906, the average temperature has increased by 0.74 °C, and this trend is projected to intensify during the next hundred years (Gao et al. 2015). As global warming continues, extreme heat events are increasing globally, which is closely associated with human health and activity (Obradovich and Fowler 2017; Varghese et al. 2019; Woodward et al. 2014; Yang et al. 2019). Under these circumstances, studies have begun to give attention to the influence of extreme temperature on emergency room visits and hospital admissions for various kinds of patients, including cardiovascular and respiratory diseases (Guo et al. 2018; Ma et al. 2019; Mohammadi et al. 2019; Watson et al. 2019). However, cataract patient has a lack of consideration.

Recently, researchers have noticed the possible relationship between high temperature and the incidence of cataract (Johnson 2004; Prokofyeva et al. 2013). Both epidemiological and laboratory evidence have shown that living in regions with high ambient temperature is related to the high prevalence of nuclear cataract (Heys et al. 2007; Prokofyeva et al. 2013; Sasaki et al. 2002; Tenkate et al. 2019). Furthermore, the dehydration crisis as a major risk factor for blinding cataract often occurs during hot weather (Johnson 2004; Minassian et al. 1989). Additionally, a previous study noticed a rise in cataract after a very hot dry summer 1 year in Iowa (Gurung et al. 2016). However, to the best of our knowledge, few studies have investigated the relationship between extreme heat and hospital admissions for cataract patients. Thus, we conducted this time-series study to investigate the association between extreme heat and hospital admissions for cataract in Hefei, China, with consideration of cumulative and lagged effects.

## Materials and methods

### Study area

**Fig. 1:**
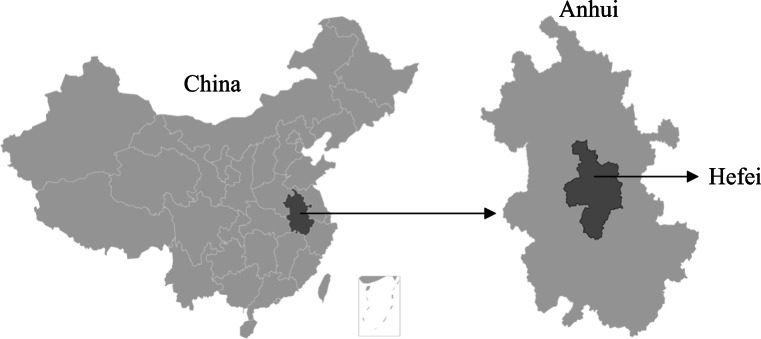
The location of Hefei in China

Hefei (31° 52′ N, 117° 17′ E), the capital of Anhui province, is located in the south-central part of China (Fig. 1). It has a resident population of over 8.1 million and an area of 11,445.1 km^2^ by 2018. Most importantly, Hefei features a subtropical humid monsoon climate and is known as one of the “Oven cities” in China. Extreme heat is a common extreme event in this city during the summer season. Taking these characteristics into account, Hefei can be regarded as an appropriate city for our research.

### Data collection

We obtained daily hospital admissions data between January 2013 and December 2015 from the New Rural Cooperative Medical System (NRCMS) provided by Anhui Health and Family Planning Commission. The NRCMS is a medical insurance scheme designed to decrease the financial burden on health spending in rural China, which covers more than 98% of rural residents (Shen et al. 2019; Xie et al. 2019a). All admission records from hospitals located in Hefei city were collected. The admission record included patient’s gender, age, date of admission, principal diagnosis, and hospital level. Diagnoses were encoded according to the International Statistical Classification of Diseases (ICD-10) and Diagnosis-related Groups (DRGs) in Hefei, Anhui. So, we identified cataract hospitalizations based on DRG codes ZDZ002 and ZDZ441, and ICD-10 codes H25–H26. Finally, our target population included those patients who had cataract hospitalizations records in Hefei between January 2013 and December 2015 in NRCMS.

Besides, daily meteorological data were collected from the National Meteorological Information Center (http://data.cma.cn). The variables included daily mean temperature and sunshine duration. Specifically, sunshine duration is the total hours in a given period during which direct solar irradiance exceeds 120 W/m^2^ (World Meteorological Organization. 2008). It accounts for the cloudiness, and thus differs from the total energy delivered by sunlight (Gu et al. 2019; Zhang et al. 2020). We combined these two datasets to conduct our main regression analyses.

### Statistical analyses

In our initial exploratory analyses, we employed a generalized additive model (GAM) to examine the exposure-response relationship between ambient temperature and the number of daily hospital admissions, with a quasi-Poisson distribution to account for over-dispersion in daily cataract cases (Lin et al. 2018; Peng et al. 2017). We chose degrees of freedom for the splines function based on generalized cross validation (GCV) value (Peng et al. 2017; Wang et al. 2012). The model was formulated as follows:

1 $$ \log \left[E\left({Y}_t\right)\right]=\alpha +s\left({\mathrm{t}\mathrm{em}}_{t-l},\mathrm{df}\right)+s\left(\mathrm{time},\mathrm{df}\right)+s\left({\mathrm{ssd}}_{\mathrm{t}},\mathrm{df}\right)+{\beta}_1{\mathrm{DOW}}_t+{\beta}_2{\mathrm{PH}}_t $$

where *t* is the day of observation; *E*(*Y*_*t*_) denotes the expected daily hospital admissions for cataract on day *t*; *α* is the intercept; *s* is a thin plate penalized spline function and *s*(tem_*t-l*_, df) means the mean temperature lagged *l* days with 5 degrees of freedom (df); time is included to control long-term trends and seasonality with 7 degrees of freedom per year (Lee et al. 2016; Peng et al. 2006; Peng et al. 2017); besides day of week (DOW_*t*_) and public holiday (PH_*t*_), we also included the nonlinear terms of sunshine duration (ssd_*t*_) with 3 degrees of freedom in this model to control solar irradiation, which is closely related to the incidence of cataract (Tenkate et al. 2019). Furthermore, as the results may vary with the specification of parameters, we also varied the df (6–9 per year) for time and changed the df (4–6) for sunshine duration in sensitivity analyses (Cheng et al. 2014; Peng et al. 2017).

According to the results of GAM, a distributed lag nonlinear model (DLNM) was applied to investigate the lagged and cumulative effects of extreme heat in the summer season (Gasparrini 2014). We also set parameters of cross-basis and splines function based on GCV value. We created the cross-basis function for temperature by specifying that its effect was null up to a high threshold and then linear based on exploratory analyses (Liu et al. 2019). Regarding the lag dimension, we set 2 degrees of freedom for the natural cubic spline. Since the lag structure in the effects of high temperature on cataract hospitalizations is unknown, we used a long period (21 days) to completely capture the lag pattern (Sun et al. 2016; Xie et al. 2019a). Summer was defined as June–August in this study (Allen and Sheridan 2018). The model in our seasonal analyses can be specified as:

2 $$ \log \left[E\left({Y}_t\right)\right]=\alpha +{\beta}_1{\mathrm{TEM}}_{t,l}+s\left(\mathrm{dos}, df\right)+{\beta}_2{\mathrm{Year}}_t+s\left({\mathrm{ssd}}_t,\mathrm{df}\right)+{\beta}_3{\mathrm{DOW}}_t+{\beta}_4{\mathrm{PH}}_t $$

where TEM_*t,l*_ is the cross-basis function for mean temperature obtained by DLNM and *l* indicates the lag days; dos (day of the season) with 4 degrees of freedom is used to control seasonality within each year, and Year_*t*_ is an indicator variable for year to describe the long-term trend (Chen et al. 2019; Yang et al. 2019; Zhang et al. 2017); other terms are the same as Eq. 1. To examine the robustness of the results of DLNM, we changed the df (3–6) for dos and altered the df (4–6) for sunshine duration.

Additionally, we stratified our analyses by gender (male and female), age group (< 65 years and ≥ 65 years), and hospital level (provincial level and other levels) to explore the sensitive population for the association between extreme heat and hospitalizations for cataract. Then, we tested the statistical significance of the differences between subgroup-specific effects through the following equation:

3 $$ Z=\left({E}_1-{E}_2\right)/\sqrt{\mathrm{SE}{\left({E}_1\right)}^2+\mathrm{SE}{\left({E}_2\right)}^2} $$

where *Z* means the *Z* test; *E*_1_ and *E*_2_ denote the effect estimates of two subgroups with corresponding standard errors SE(*E*_1_) and SE(*E*_2_) (Altman and Bland 2003; Chen et al. 2019; Yang et al. 2019).

We summarized the results by computing the relative risks and their 95% confidence intervals. The details about the calculation were mentioned in previous studies (Gasparrini 2011; Gasparrini et al. 2010). RR > 1 denotes that mean temperature is a risk factor and RR < 1 indicates that mean temperature is a protective factor; RR = 1 suggests that mean temperature is not related to cataract hospitalizations (Gutiérrez-Torres 2020). Our multivariate analyses were conducted in R software, version 3.5.2, using the “mgcv” and “dlnm” packages. The statistical tests were two-sided, and *p* < 0.05 was considered statistically significant.

## Results

### Data description

As shown in Table 1, there were a total of 26,123 hospital admissions for cataract from 2013 to 2015 in our study, including 10,220 (39.12%) male cases and 15,903 (60.88%) female cases. These patients were predominantly greater than or equal to 65 years old (75.70%), and a large percentage (73.58%) of them were not admitted to provincial-level hospitals.

**Table 1:** Demographic characteristics of daily hospital admissions for cataract in Hefei, China, 2013–2015

	*N*	%	Proportion test^a^
Total	26,123	100	
Gender			
Male	10,220	39.12	*p* < 0.01
Female	15,903	60.88	
Age			
< 65	6349	24.30	*p* < 0.01
≥ 65	19,774	75.70	
Hospital level			
Provincial level	6903	26.42	*p* < 0.01
Other levels	19,220	73.58	

^a^The proportion test (Newcombe 1998; Verzani 2018) was used to examine the statistical differences between hospital admissions for cataract in different groups

**Fig. 2:**
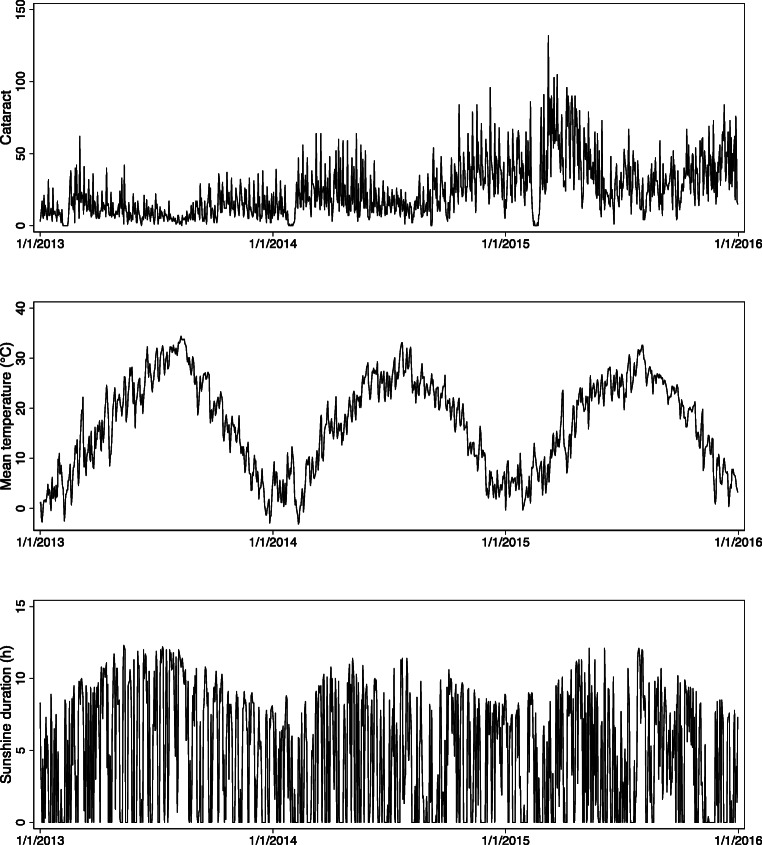
The time-series distribution of cataract hospitalization and meteorological factors in Hefei, China, 2013–2015

Summary statistics for the daily number of cataract admission and weather factors during the study period are summarized in Table 2. And Fig. 2 plots the time-series distribution of their variations. Average daily mean temperature and sunshine duration were 16.78 °C and 4.61 h, respectively. According to the Spearman correlation analysis, we found that daily mean temperature had a positive correlation with sunshine duration (*r* = 0.23, *p* < 0.05), which was not strong.

**Table 2:** Summary statistics for the daily number of cataract admission and meteorological factors in Hefei, China, 2013–2015

Variables	Mean (SD)	Min	Percentile			Max
			25	50	75	
Daily hospital admissions	23.86 (19.52)	0	9	19	33	132
Mean temperature (°C)	16.78 (9.25)	− 3.1	8.5	18.1	24.6	34.4
Sunshine duration (h)	4.61 (4.07)	0	0	4.7	8.3	12.3

### Regression results

**Fig. 3:**
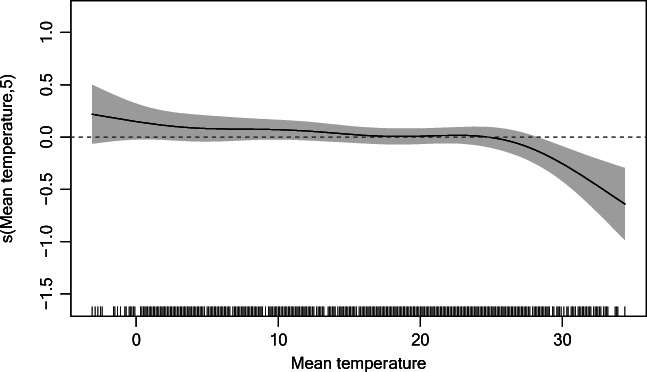
Relationship between current mean temperature and hospital admissions for cataract. The *x* axis is the mean temperature. The *y* axis indicates the contribution of the smoother to the fitted values

Figure 3 plots the results of our exploratory analysis. The exposure-response relationship for current mean temperature and cataract admissions was approximately linear above 28 °C (the 90th percentile of daily mean temperature). Similar relationships were obtained from different lag periods of mean temperature (Fig. S1). Consequently, 28 °C was considered the threshold to quantify the effect of extreme heat in DLNM. These results were robust in our sensitivity analyses (Fig. S2).

**Fig. 4:**
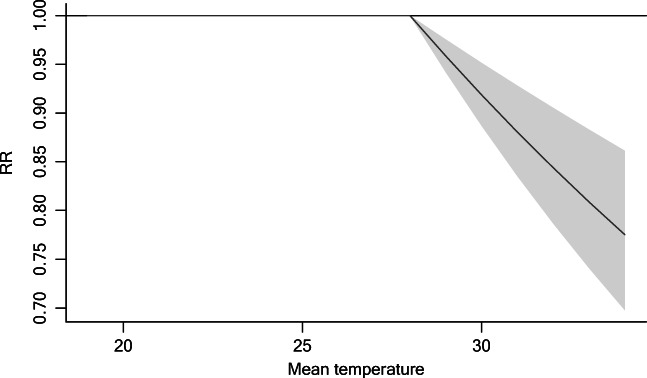
Relative risk of current day temperature on cataract hospitalizations

**Fig. 5:**
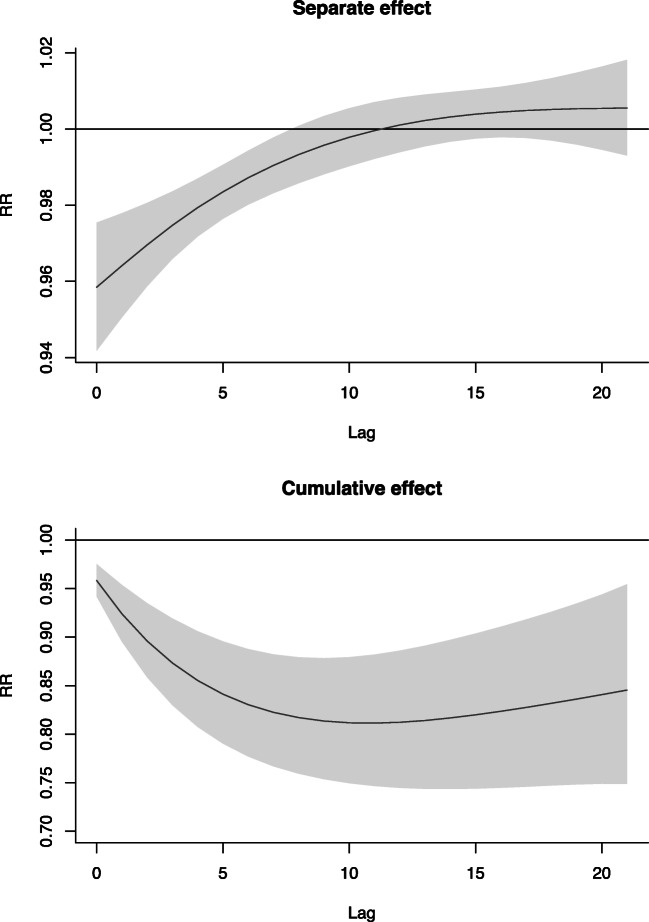
Separate and cumulative effects of mean temperature (for 1 °C beyond the threshold) on cataract hospitalizations

Figure 4 and Fig. 5 show the results of DLNM. In summer, mean temperature was significantly associated with hospital admissions for cataract at lag 0 day. When daily mean temperature was above 28 °C, each 1 °C rise was associated with a 4% decrease in the number of cataract admissions (RR = 0.96, 95% CI = 0.94–0.98). After lag 8 days, the separate lag effect became statistically insignificant. The cumulative relative risk over 11 days of lag was the lowest, which indicated that every 1 °C increase in mean temperature above 28 °C was associated with a 19% decrease in the number of hospital admissions for cataract (RR = 0.81, 95% CI = 0.75–0.88). Similar results were obtained when changing the df for dos and sunshine duration (Fig. S3–S4).

Table 3 presents the relative risks at lag 0 day and cumulative relative risks at lag 0–11 days for the associations of extreme heat with hospitalizations for cataract in each subgroup. We found that for every 1 °C increase in temperature above 28 °C, the reduction of hospital admissions for males (Lag 0: RR = 0.95, 95% CI = 0.93–0.97; Lag 0–10: RR = 0.82, 95% CI = 0.73–0.91) and females (Lag 0: RR = 0.96, 95% CI = 0.94–0.98; Lag 0–10: RR = 0.81, 95% CI = 0.73–0.89) in summer is similar. For the age-specific effects, we observed slightly lower relative risks for the elderly (≥ 65 years) (Lag 0: RR = 0.95, 95% CI = 0.93–0.97; Lag 0–10: RR = 0.78, 95% CI = 0.70–0.87). But the difference was statistically insignificant according to *Z* test (*p* > 0.05). For patients who were admitted to provincial-level hospitals, their hospitalizations were insignificantly associated with extreme high temperature at lag 0 (RR = 0.98, 95% CI = 0.96–1.00). And the differences between the hospital level-specific effects were statistically significant (Lag 0: *p* < 0.05; Lag 0–10: *p* < 0.1).

**Table 3:** Relative risks of mean temperature (for 1 °C beyond the threshold) on hospital admissions for cataract in different subgroups

	Lag 0		Lag 0–11	
	RR (95% CI)	*Z* test	Cumulative RR (95% CI)	*Z* test
Gender				
Male	0.95 (0.93–0.97)	*p* > 0.1	0.82 (0.73–0.91)	*p* > 0.1
Female	0.96 (0.94–0.98)		0.81 (0.73–0.89)	
Age				
< 65	0.97 (0.95–0.99)	*p* > 0.1	0.87 (0.78–0.96)	*p* > 0.1
≥ 65	0.95 (0.93–0.97)		0.78 (0.70–0.87)	
Hospital level				
Provincial level	0.98 (0.96–1.00)	*p* < 0.05	0.88 (0.80–0.97)	*p* < 0.1
Other levels	0.94 (0.92–0.97)		0.77 (0.69–0.87)	

## Discussion

In this paper, we explored the association between ambient temperature and cataract hospitalizations by using a generalized additive model and a distributed lag nonlinear model. Our results showed that extreme heat was negatively associated with hospital admissions for cataract. However, previous studies found that high temperature was a risk factor for the incidence of cataract, contrary to our finding (Heys et al. 2007; Prokofyeva et al. 2013; Sasaki et al. 2002).

The underlying mechanisms of our different finding can be explained in two aspects. Firstly, our results could be caused by the reduction in personal ultraviolet radiation exposure during high temperature, which is related to the incidence of cataract (Tenkate et al. 2019). A recent study in the USA observed that extreme heat could reduce recreational physical activity, mainly because it is too hot to go outdoors (Obradovich and Fowler 2017). From this point of view, extreme high temperature could make people avoid staying in the sun, consequently getting less ultraviolet rays. Besides, to minimize heat stress, outdoor workers also choose to reduce activities when temperature is high, thus leading to less occupational exposure to solar ultraviolet radiation (Yi and Chan 2015). A previous investigation also showed that personal ultraviolet radiation exposure of participants measured with dosimeters is higher in spring than in hot summer (Sun et al. 2014).

Second, our finding could also be caused by the reduction of hospital-related activities in extreme heat. Cataract patients usually worry that the treatment effect on acutely hot days may be worse than that during comfortable weather since extreme heat could increase the risk of infection for them after surgical treatment (Anthony et al. 2017; Rubio 2004). For example, when average monthly temperature increases 2.8 °C, the odds of the admission caused by primary surgical site infections increase 2.1%; specifically, compared with temperatures less than 4.4 °C, temperature larger than 32.2 °C is related to an increase in the odds of this admission of 28.9% (Anthony et al. 2017). Thus, cataract patients may not prefer to accept treatment on hot days, causing fewer hospital admissions. Furthermore, a study exactly observed that the number of cataract admissions and phacoemulsification surgeries decreased during the summer (Leong et al. 2006). On the other hand, previous studies found that patients were less likely to visit hospitals during bad weather (Lee et al. 2016; Ou et al. 2005; Xie et al. 2019b). So, as a kind of severe weather condition, extreme heat could also prevent cataract patients from visiting doctors since their condition is usually not urgent, thus leading to fewer cataract admissions.

In subgroup analyses, the negative association between extreme heat and hospital admissions for cataract was stronger among patients who were not admitted to provincial-level hospitals. It may be caused by the competition for limited medical resources, like bed availability (Kc and Terwiesch 2017; Keegan 2010). In China, getting a chance to be admitted to provincial-level hospitals is generally difficult for most patients. For instance, patients may wait several days or weeks for a bed. Therefore, it is not reasonable for people to forgo the admission opportunity just because of the hot weather.

Our study has several implications. First, we found a negative association between extreme heat and cataract hospitalizations, which is useful for hospitals to adjust medical resources during hot weather. Second, besides morbidity, other mechanisms (like the treatment quality) could also affect the association between weather and hospitalizations. Cataract patients may worry about the treatment effect during hot weather, which should be noticed and improved. And our findings could add new evidence that patients are less likely to visit doctors during bad weather.

However, some limitations should be acknowledged. The ambient temperature may be different from the true exposure of patients leading to under-evaluation of solar UVR role, which could raise exposure measurement errors (Ge et al. 2018; Niu et al. 2016). Additionally, as we only considered rural patients in Hefei, more works are needed to check whether our findings can be replicated for urban people and other cities. Last, there are unobserved patient and hospital characteristics, healthcare behaviors which could be confounding factors.

## Conclusion

To the best of our knowledge, this is the first study to explore the association between ambient temperature and hospital admissions for cataract patients. We found that extreme heat was negatively associated with cataract hospitalizations, providing useful implications for hospitals to manage medical resources during hot weather. Further studies are needed to examine the relationship in other populations or other cities.

## Electronic supplementary material

ESM 1 (DOCX 763 kb)

## References

[CR1] Allen MJ, Sheridan SC (2018). Mortality risks during extreme temperature events (ETEs) using a distributed lag non-linear model. Int J Biometeorol.

[CR2] Altman DG, Bland JM (2003). Interaction revisited: the difference between two estimates. BMJ.

[CR3] Anderson DF, Dhariwal M, Bouchet C, Keith MS (2018). Global prevalence and economic and humanistic burden of astigmatism in cataract patients: a systematic literature review. Clin Ophthalmol (Auckland, NZ).

[CR4] Anthony CA, Peterson RA, Polgreen LA, Sewell DK, Polgreen PM (2017). The seasonal variability in surgical site infections and the association with warmer weather: a population-based investigation. Infect Control Hosp Epidemiol.

[CR5] Chen J, Yang J, Zhou M, Yin P, Wang B, Liu J, Chen Z, Song X, Ou C-Q, Liu Q (2019). Cold spell and mortality in 31 Chinese capital cities: definitions, vulnerability and implications. Environ Int.

[CR6] Cheng J, Wu J, Xu Z, Zhu R, Wang X, Li K, Wen L, Yang H, Su H (2014). Associations between extreme precipitation and childhood hand, foot and mouth disease in urban and rural areas in Hefei, China. Sci Total Environ.

[CR7] Delavar A, Freedman DM, Velazquez-Kronen R, Little MP, Kitahara CM, Alexander BH, Linet MS, Cahoon EK (2018). Ultraviolet radiation and incidence of cataracts in a nationwide US cohort. Ophthalmic Epidemiol.

[CR8] Gao J, Sun Y, Liu Q, Zhou M, Lu Y, Li L (2015). Impact of extreme high temperature on mortality and regional level definition of heat wave: a multi-city study in China. Sci Total Environ.

[CR9] Gasparrini A (2011) Distributed lag linear and non-linear models in R: the package dlnm. J Stat Softw 43:1 https://www.ncbi.nlm.nih.gov/pmc/articles/PMC3191524/. Accessed 8 July 2020 PMC3191524 22003319

[CR10] Gasparrini A (2014). Modeling exposure–lag–response associations with distributed lag non-linear models. Stat Med.

[CR11] Gasparrini A, Armstrong B, Kenward MG (2010). Distributed lag non-linear models. Stat Med.

[CR12] Ge Y, Liu C, Niu Y, Chen C, Wang W, Lin Z, Chen R, Cai J, Kan H (2018). Associations between ambient temperature and daily hospital admissions for rheumatic heart disease in Shanghai, China. Int J Biometeorol.

[CR13] Gu S, Huang R, Yang J, Sun S, Xu Y, Zhang R, Wang Y, Lu B, He T, Wang A (2019). Exposure-lag-response association between sunlight and schizophrenia in Ningbo, China. Environ Pollut.

[CR14] Guo Y, Ma Y, Ji J, Liu N, Zhou G, Fang D, Huang G, Lan T, Peng C, Yu S (2018). The relationship between extreme temperature and emergency incidences: a time series analysis in Shenzhen, China. Environ Sci Pollut Res.

[CR15] Gurung D, Gokul K, Adhikary P (2016). Mathematical model of thermal effects of blinking in human eye. Int J Biomath.

[CR16] Gutiérrez-Torres JD (2020). Temporal lagged relationship between a vegetation index and cutaneous leishmaniasis cases in Colombia: an analysis implementing a distributed lag nonlinear model. Parasitol Res.

[CR17] Heys KR, Friedrich MG, Truscott RJ (2007). Presbyopia and heat: changes associated with aging of the human lens suggest a functional role for the small heat shock protein, α-crystallin, in maintaining lens flexibility. Aging Cell.

[CR18] Johnson G (2004). The environment and the eye. Eye.

[CR19] Kc DS, Terwiesch C (2017). Benefits of surgical smoothing and spare capacity: an econometric analysis of patient flow. Prod Oper Manag.

[CR20] Keegan AD (2010). Hospital bed occupancy: more than queuing for a bed. Med J Aust.

[CR21] Lee HJ, Jin MH, Lee JH (2016). The association of weather on pediatric emergency department visits in Changwon, Korea (2005–2014). Sci Total Environ.

[CR22] Leong AM, Crighton EJ, Moineddin R, Mamdani M, Upshur RE (2006). Time series analysis of age related cataract hospitalizations and phacoemulsification. BMC Ophthalmol.

[CR23] Lin H, Tao J, Kan H, Qian Z, Chen A, Du Y, Liu T, Zhang Y, Qi Y, Ye J (2018). Ambient particulate matter air pollution associated with acute respiratory distress syndrome in Guangzhou, China. J Expo Sci Environ Epidemiol.

[CR24] Liu Z, Liu Y, Zhang Y, Lao J, Zhang J, Wang H, Jiang B (2019). Effect of ambient temperature and its effect modifiers on bacillary dysentery in Jinan, China. Sci Total Environ.

[CR25] Ma Y, Zhou J, Yang S, Yu Z, Wang F, Zhou J (2019). Effects of extreme temperatures on hospital emergency room visits for respiratory diseases in Beijing, China. Environ Sci Pollut Res.

[CR26] Minassian D, Mehra V, Verrey J (1989). Dehydrational crises: a major risk factor in blinding cataract. Br J Ophthalmol.

[CR27] Modenese A, Gobba F (2018). Cataract frequency and subtypes involved in workers assessed for their solar radiation exposure: a systematic review. Acta Ophthalmol.

[CR28] Mohammadi D, Zare Zadeh M, Zare Sakhvidi MJ (2019): Short-term exposure to extreme temperature and risk of hospital admission due to cardiovascular diseases. Int J Environ Health Res, 1–11. 10.1080/09603123.2019.1663496 10.1080/09603123.2019.1663496 33615930

[CR29] Newcombe RG (1998). Two-sided confidence intervals for the single proportion: comparison of seven methods. Stat Med.

[CR30] Niu Y, Chen R, Liu C, Ran P, Chen A, Chen X, Kan H (2016). The association between ambient temperature and out-of-hospital cardiac arrest in Guangzhou, China. Sci Total Environ.

[CR31] Obradovich N, Fowler JH (2017). Climate change may alter human physical activity patterns. Nat Hum Behav.

[CR32] Ou DK, To TP, Taylor DM (2005). Weather patients will come?. Med J Aust.

[CR33] Pascolini D, Mariotti SP (2012). Global estimates of visual impairment: 2010. Br J Ophthalmol.

[CR34] Peng RD, Dominici F, Louis TA (2006). Model choice in time series studies of air pollution and mortality. J Roy Stat Soc Ser A (Stat Soc).

[CR35] Peng Z, Wang Q, Kan H, Chen R, Wang W (2017). Effects of ambient temperature on daily hospital admissions for mental disorders in Shanghai, China: a time-series analysis. Sci Total Environ.

[CR36] Prokofyeva E, Wegener A, Zrenner E (2013). Cataract prevalence and prevention in Europe: a literature review. Acta Ophthalmol.

[CR37] Rubio EF (2004). Climatic influence on conjunctival bacteria of patients undergoing cataract surgery. Eye.

[CR38] Sasaki H, Jonasson F, Shui Y, Kojima M, Ono M, Katoh N, Cheng H-M, Takahashi N, Sasaki K (2002): High prevalence of nuclear cataract in the population of tropical and subtropical areas, Progress in lens and cataract research. Karger Publishers, pp. 60–69 10.1159/000060806 12061279

[CR39] Shen X, Chai J, Xiao S, Yao A, Wen K, Pan Q, Wang D (2019). Effect of insurance-reimbursed inpatient cancer care in Anhui Province: a retrospective study. Lancet.

[CR40] Song P, Wang H, Theodoratou E, Chan KY, Rudan I (2018). The national and subnational prevalence of cataract and cataract blindness in China: a systematic review and meta-analysis. J Glob Health.

[CR41] Sun J, Lucas R, Harrison S, van der Mei I, Armstrong BK, Nowak M, Brodie A, Kimlin MG (2014). The relationship between ambient ultraviolet radiation (UVR) and objectively measured personal UVR exposure dose is modified by season and latitude. Photochem Photobiol Sci.

[CR42] Sun S, Tian L, Qiu H, Chan K-P, Tsang H, Tang R, Lee RS-Y, Thach T-Q, Wong C-M (2016). The influence of pre-existing health conditions on short-term mortality risks of temperature: evidence from a prospective Chinese elderly cohort in Hong Kong. Environ Res.

[CR43] Tenkate T, Adam B, Al-Rifai RH, Chou BR, Gobba F, Ivanov ID, Leppink N, Loney T, Pega F, Peters CE (2019). WHO/ILO work-related burden of disease and injury: protocol for systematic reviews of occupational exposure to solar ultraviolet radiation and of the effect of occupational exposure to solar ultraviolet radiation on cataract. Environ Int.

[CR44] Varghese BM, Hansen A, Nitschke M, Nairn J, Hanson-Easey S, Bi P, Pisaniello D (2019). Heatwave and work-related injuries and illnesses in Adelaide, Australia: a case-crossover analysis using the Excess Heat Factor (EHF) as a universal heatwave index. Int Arch Occup Environ Health.

[CR45] Verzani J (2018): Using R for introductory statistics. CRC press 10.1201/9781315373089

[CR46] Wang X-L, Yang L, Chan K-P, Chiu SS, Chan K-H, Peiris JM, Wong C-M (2012). Model selection in time series studies of influenza-associated mortality. PLoS One.

[CR47] Watson KE, Gardiner KM, Singleton JA (2019) The impact of extreme heat events on hospital admissions to the Royal Hobart Hospital. J Public Health. 10.1093/pubmed/fdz033 10.1093/pubmed/fdz033 31220305

[CR48] Woodward A, Smith KR, Campbell-Lendrum D, Chadee DD, Honda Y, Liu Q, Olwoch J, Revich B, Sauerborn R, Chafe Z (2014). Climate change and health: on the latest IPCC report. Lancet.

[CR49] World Meteorological Organization (2008): Guide to Meteorological Instruments and Methods of Observation (Seventh edition) https://www.webcitation.org/6E9CzPWoA?url=http://www.wmo.int/pages/prog/gcos/documents/gruanmanuals/CIMO/CIMO_Guide-7th_Edition-2008.pdf. Accessed 7 July 2020

[CR50] Xie J, Teng J, Fan Y, Xie R, Shen A (2019). The short-term effects of air pollutants on hospitalizations for respiratory disease in Hefei, China. Int J Biometeorol.

[CR51] Xie J, Zhu Y, Fan Y, Xin L, Liu J (2019b) Association between rainfall and readmissions of rheumatoid arthritis patients: a time-stratified case-crossover analysis. Int J Biometeorol. 10.1007/s00484-019-01805-y 10.1007/s00484-019-01805-y 31650297

[CR52] Yang J, Yin P, Sun J, Wang B, Zhou M, Li M, Tong S, Meng B, Guo Y, Liu Q (2019). Heatwave and mortality in 31 major Chinese cities: definition, vulnerability and implications. Sci Total Environ.

[CR53] Yi W, Chan AP (2015). Optimal work pattern for construction workers in hot weather: a case study in Hong Kong. J Comput Civ Eng.

[CR54] Zhang X, Wang Y, Chen X, Zhang X (2020). Associations between prenatal sunshine exposure and birth outcomes in China. Sci Total Environ.

[CR55] Zhang Y, Feng R, Wu R, Zhong P, Tan X, Wu K, Ma L (2017). Global climate change: impact of heat waves under different definitions on daily mortality in Wuhan, China. Global Health Res Policy.

