# Supplementary material for: Association between extreme heat and hospital admissions for cataract patients in Hefei, China
Source: Environ Sci Pollut Res Int. 2020 Aug 13;27(36):45381–9. doi: 10.1007/s11356-020-10402-1 (PMC7686207; doi:10.1007/s11356-020-10402-1)
Supplement: Supplementary file 1 — (DOCX 763 kb) [file 11356_2020_10402_MOESM1_ESM.docx]

**Fig. S1** Relationship between hospital admissions for cataract and mean temperature during different lag periods

**Fig. S2** Relationship between current mean temperature and hospital admissions for cataract when changing the *df* for *time* and sunshine duration

**Fig. S3** Separate effects of mean temperature on cataract hospitalizations when changing the *df* for *dos* and sunshine duration

**Fig. S4** Cumulative effects of mean temperature on cataract hospitalizations when changing the *df* for *dos* and sunshine duration
